# A computational model for how cells choose temporal or spatial sensing during chemotaxis

**DOI:** 10.1371/journal.pcbi.1005966

**Published:** 2018-03-05

**Authors:** Rui Zhen Tan, Keng-Hwee Chiam

**Affiliations:** 1 Singapore Institute of Technology, Singapore; 2 Bioinformatics Institute, A*STAR, Singapore; Duke University, UNITED STATES

## Abstract

Cell size is thought to play an important role in choosing between temporal and spatial sensing in chemotaxis. Large cells are thought to use spatial sensing due to large chemical difference at its ends whereas small cells are incapable of spatial sensing due to rapid homogenization of proteins within the cell. However, small cells have been found to polarize and large cells like sperm cells undergo temporal sensing. Thus, it remains an open question what exactly governs spatial versus temporal sensing. Here, we identify the factors that determines sensing choices through mathematical modeling of chemotactic circuits. Comprehensive computational search of three-node signaling circuits has identified the negative integral feedback (NFB) and incoherent feedforward (IFF) circuits as capable of adaptation, an important property for chemotaxis. Cells are modeled as one-dimensional circular system consisting of diffusible activator, inactivator and output proteins, traveling across a chemical gradient. From our simulations, we find that sensing outcomes are similar for NFB or IFF circuits. Rather than cell size, the relevant parameters are the 1) ratio of cell speed to the product of cell diameter and rate of signaling, 2) diffusivity of the output protein and 3) ratio of the diffusivities of the activator to inactivator protein. Spatial sensing is favored when all three parameters are low. This corresponds to a cell moving slower than the time it takes for signaling to propagate across the cell diameter, has an output protein that is polarizable and has a local-excitation global-inhibition system to amplify the chemical gradient. Temporal sensing is favored otherwise. We also find that temporal sensing is more robust to noise. By performing extensive literature search, we find that our prediction agrees with observation in a wide range of species and cell types ranging from *E*. *coli* to human Fibroblast cells and propose that our result is universally applicable.

## Author summary

Unicellular organisms and other single cells often have to migrate towards food sources or away from predators by sensing chemicals present in the environment. There are two ways for a cell to sense these external chemicals: temporal sensing, where the cell senses the external chemical at two different time points after it has moved through a certain distance, or spatial sensing, where the cell senses the external chemical at two different locations on its cellular surface (e.g., the front and rear of the cell) simultaneously. It has been thought that small unicellular organisms employ temporal sensing as their small size prohibits sensing at two different locations on the cellular surface. Using computational modeling, we find that the choice between temporal and spatial sensing is determined by the ratio of cell velocity to the product of cell diameter and rate of signaling, as well as the diffusivities of the signaling proteins. Predictions from our model agree with experimental observations over a wide range of cells, where a fast-moving, small cell performs better comparing the chemoattractant at different times in its trajectory; whereas, a slow-moving, big cell performs better by comparing the chemoattractant concentration at its two ends.

## Introduction

**Fig 1:**
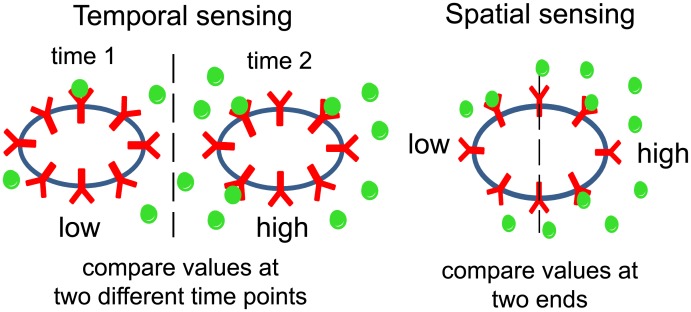
Schematic of temporal and spatial sensing. For temporal sensing, the cell compares the output at two different time points (left). For spatial sensing, the cell compares the ouput at its two ends (right).

Chemotaxis is the process whereby cells move towards a region of higher chemical stimulus concentration. Cellular movements towards the favorable direction enables, for example, prokaryotic unicellular organisms such as *Escherichia* *coli* (*E*. *coli*) to move towards food and eukaryotic cells such as neutrophils and macrophages to move towards the site of infection to phagocytize external parasites. Information about the external chemical gradient is transduced into the cell by binding of chemoattractant and chemorepellant molecules to specific receptors at the cell surface. These binding events then trigger downstream intracellular signaling to modulate the cell’s motility. To move up or down the gradient, cells can adopt two distinct strategies: temporal sensing or spatial sensing (Fig 1). In temporal or sequential sensing, cells compare the intensity of receptor stimulation at different times (Fig 1, left) and modulate their probability of moving in the same direction or switching directions. In *E*. *coli*, an organism exhibiting temporal sensing, rotation of its flagella in the counter-clockwise direction results in directed motion whereas rotation in the clockwise direction results in tumbling and a random change in direction [1, 2]. Binding of chemoattractant decreases the switching probability from counter-clockwise to clockwise rotation, thus reducing tumbling and increasing the run length when the cell is moving in the favorable direction. In spatial sensing, cells simultaneously measure the intensity of receptor stimulation at its two ends (Fig 1, right). The different receptor stimulation leads to cell polarization and motility in the preferred direction. In neutrophils, G protein-coupled receptors (GPCRs) are originally evenly distributed along the plasma membrane. Binding of chemoattractant results in activation of signaling pathways involving small Rho guanosine triphosphatases (Rho GTPases) and phosphoinositide 3 kinases (PI3Ks) and asymmetric polymerization of actin at the up-gradient edge of the cell, facilitating motion up the gradient [3].

The decision whether to employ temporal or spatial sensing has largely been attributed to cell size. It is thought that large cells have an advantage for spatial sensing as the intensities of receptor stimulation are expected to be very different at its two ends. In contrast, small cells of around or less than a micron in diameter are unable to exhibit spatial sensing as chemical gradients are rapidly homogenized by fast diffusion. For example, in an *E*. *coli* of 2*um*, the cytoplasmic CheY chemotaxis signal transduction protein with a diffusion constant of 4.6±0.8*um*^2^*s*^−1^[4] will take only 0.9*s* to transerve the cell. However, spatial localization of MinC, MinD and MinE proteins to bring about proper cell division [5] and polar localization of the chemoreceptor complex of cytoplasmic CheA and CheW proteins [6] suggest that spatial segregration of proteins can be established at the micron scale in small cells. Berg and Purcell also showed theoretically that, in principle, an immobile *E*. *coli* cell is able to perform spatial sensing [7]. Dusenbery, based on arguments of signal-to-noise ratio, also found that the cell size limit for spatial sensing (< 1*um*) is close to that for temporal sensing and is actually smaller than the size of many prokaryotes [8]. These works cast doubts on previous arguments for the inability of small cells of around a micron in diameter to perform spatial sensing and suggests that most cells, whether big and small, are able to perform both spatial and temporal sensing. Here, we use computational model to show that the decision to perform either temporal or spatial sensing is instead determined by the performance of each type of sensing. To determine the performance of temporal and spatial sensing, we need to integrate the sensing mechanism with the network circuits use for chemotaxis.

**Fig 2:**
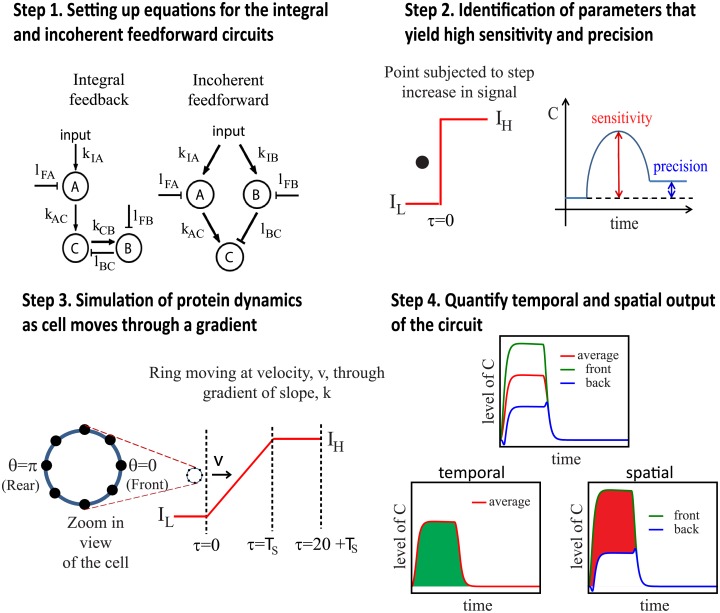
Steps in the computational modelling of network motifs.

A key goal in systems biology is to identify network motifs capable of achieving certain biological function. For chemotaxis to be effective, cells need to exhibit adaptation. Adaptation refers to a cell’s ability to respond to a change in the input stimulus and then return to its original level, even when the input stimulus remains high. This property allows cells to respond to a high range of chemoattractant concentration. Extensive efforts to understand the ability of *E*. *coli* to remain sensitive to a wide range of chemoattractant has led to the identification of the negative integral feedback (NFB) circuit for chemotaxis [9, 10] (Fig 2, step 1, left). In NFB, following stimulation of the output protein (protein *C*) by the activator (protein *A*), a buffering component/inactivator (protein *B*) integrates the difference between the response and the baseline level and feeds this difference back into the response, enabling the output protein to return to the basal level after each pulse of chemoattractant. On the other hand, modeling efforts in eukaryotic gradient sensing have identified the incoherent feedforward (IFF) circuit (Fig 2, step 1, right) for amplification of the signaling response to shallow gradients [11–13]. In IFF, two nodes, an activator (protein *A*) and a repressor (protein *B*), are activated proportionally to the stimulus but act with opposite effects on the output protein (protein *C*). Like the NFB, the IFF circuit also has the adaptive property needed for sensing a wide range of chemoattractant. A comprehensive survey of all possible three-node network topologies had been carried out to search for networks that yield biochemical adaptation response [14]. They found that minimal circuits containing NFB and IFF motifs yield adaptation and that more complicated circuits that yield adaptation contain at least one of these two motifs. Hence we will use the NFB and IFF circuits to study cells’ chemotaxic response as they are the basic building blocks for three-node circuits that can yield adaptative property, an essential property for chemotaxis.

We compare the performance of temporal and spatial sensing when a cell uses the NFB and IFF circuits by determining the conditions that favor one mode of sensing over the other. In temporal sensing, the cell compares the level of *C* with the steady state level of *C* (area highlighted in green) (Fig 2, step 4, temporal) whereas in spatial sensing, the cell compares the level of *C* at the front half and back half of the cell (area highlighted in red) (Fig 2, step 4, spatial). We identify five dimensionless terms, namely the diffusivities of the activator (protein *A*), repressor (protein *B*) and output (protein *C*) proteins, all normalised to the deactivation rate of the output protein, the effective chemoattractant gradient experienced by the moving cell, and the ratio of cell speed to the product of diameter and signaling rate that characterize the response of the negative integral feedback and incoherent feedforward circuits. By varying these five terms and comparing the performance of temporal and spatial sensing on the negative integral feedback and incoherent feedforward circuits, we find that spatial sensing performs better than temporal sensing in the regime where the cell velocity is small relative to the product of cell diameter and the circuit reaction rate, and when the repressor protein (protein *B*) diffuses faster than protein *A*) and the diffusibility of the output protein (protein *C*) is low. In all other cases, temporal sensing performs better. By incorporating noise into our analysis, we also found that temporal sensing is more robust to noise than spatial sensing.

## Results

### Chemotaxis circuits characterized by dimensionless diffusion rates, effective chemoattractant gradient and ratio of cell speed to cell diameter

Here, we want to determine whether cell size is the determining factor or there are other factors contributing to the choice between temporal and spatial sensing. We assume that the mode of sensing that yields higher signaling output will be adopted by cells. In general, the signaling output will depend on both the signaling (i.e., molecular) and physical properties of the chemotactic cell as well as the properties of the chemoattractant. Hence, we need to identify these important variables and determine how they affect the signaling outputs. However, one obstacle is that, very often, the values of these variables have not been measured experimentally. Thus, we will adopt a network motifs approach where the exact parameter values are not so critical as long as the parameter values lie within certain regimes, since the same behavior is typically observed over a range of parameter vaues. Hence, we will identify all possible behaviors of the networks by sweeping through parameter space. This approach has been widely adopted in modeling papers (e.g., Ma et al., 2014). In our analysis, we have swept through 4 to 5 orders of magnitude of parameter values and obtained the perfect adaptive behavior expected for the network motifs.

Our analysis consists of four steps (Fig 2). We first set up the equations for the negative integral (NFB) and incoherent feedforward (IFF) circuits (Fig 2, step 1) and identify 100 sets of parameters that lead to high sensitivity and adaptation precision for these circuits (Fig 2, step 2). High sensitivity is responsible for signal amplication in shallow gradients whereas high adaptation precision is required for signal adaptation. These are properties that enables a cell to perform chemotaxis effectively. Next, we determine the protein dynamics as the cell moves through a linear gradient for the sets of parameters that we have identified in step 2 for the NFB and IFF circuits (Fig 2, step 3). The cell is modeled as a one dimensional ring, of diameter *d*, with an activator protein (*A*), inactivator protein (*B*) and output protein (*C*) that can diffuse freely on the cell membrane. At time *τ* = 0, the cell moves with velocity, *v*, into a linear chemoattractant gradient with slope, *k*. The cell experiences the gradient for a fixed time, *T*_*s*_, before moving into a region with constant *I* = *I*_*H*_. The cell uses both the NFB and IFF circuits to process the chemoattractant input and interprets the results using temporal or spatial sensing (Fig 2, step 4). More details can be found in S1 Text.

Extending the equations for incoherent feedforward and negative integral feedback circuits to account for spatial differences of protein levels on the cell’s membrane and a changing external chemoattractant gradient (see S1 Text), we find that the equations are fully described by the following variables: cell diameter *d*, cell velocity *v*, chemoattractant gradient *k* (which has the unit of inverse length), signaling rates, of which we choose *l*_*BC*_, the deactivation rate of *C*, to be representative (i.e., other signaling rate can be expressed as ratios of it), and the activator (*A*), inactivator (*B*), and output protein (*C*) diffusivities, *D*_*A*_, *D*_*B*_, and *D*_*C*_, respectively. These variables can be grouped into the following five dimensionless variables below.

The five dimensionless variables are as follows:

1. $\alpha =\frac{vk}{l_{BC}}$ characterizes the ratio of the rate over which the chemoattractant varies to the signaling rate. The numerator *vk* (which has the unit of inverse time) can be interpreted as the rate at which the cell experiences changes in the chemoattractant as it moves. In general, the convective or material derivative of the chemoattractant field experienced by a moving cell is *d*/*dt* = ∂/∂*t* + *v* ⋅ ∂/∂*x*, where we assume the cell moves in one dimension (*x*) which is also the dimension of the chemoattractant slope. In our example here, the chemoattractant field is stationary (∂/∂*t* = 0) and hence *d*/*dt* = *vk*.
2. $\beta =\frac{v}{dl_{BC}}$ is the ratio of cell speed to the product of cell diameter and signaling rate. This suggests that *v* and *d* acted in opposite ways to affect the signaling profile of protein C in response to the chemoattractant gradient.
3. $D_{A}^{{}^{\prime }}=\frac{D_{A}}{d^{2}l_{BC}}$, $D_{B}^{{}^{\prime }}=\frac{D_{B}}{d^{2}l_{BC}}$ and $D_{C}^{{}^{\prime }}=\frac{D_{C}}{d^{2}l_{BC}}$ are the dimensionless diffusion coefficients. Notice that the dimensionless diffusion coefficients are the diffusion coefficients normalized by both *l*_*BC*_ and the square of the diameter of the cell. This means that for the same value of the diffusion coefficient and *l*_*BC*_, a small cell will have a higher value for the dimensionless diffusion coefficients than a big cell. This agrees qualitatively with the intuition that chemical gradients are more rapidly homogenized in small cells. Furthermore, since the time taken for a protein to transverse across the diameter is given by the square of the diameter over the diffusion coefficient, the dimensionless diffusion coefficients can also be view as the ratio of signaling time over the time taken for the protein to transverse the cell. Hence having a large dimensionless diffusion coefficient means that the protein diffuses much faster than signaling rate in the cell.

First, we consider the effect of $D_{C}^{{}^{\prime }}$ on the choice between temporal versus temporal sensing. For spatial sensing, the cell compares the level of protein *C* at different parts of the cell. Therefore, *C* has to diffuse slowly to allow for spatial sensing. When $D_{C}^{{}^{\prime }}$ is big, any spatial information will be rapidly homogenized and temporal sensing will be favored. We use $D_{C}^{{}^{\prime }}=0$ for our analysis to study the effects of other parameters on the sensing choice.

### The rate of chemoattractant variation does not govern sensing choice

We hypothesize that *α* would not affect signaling outcome as a steeper or more gentle external gradient would affect the output from both sensing choice equally. To test this hypothesis, we vary *α* = 0.00001, 0.0001, 0.001, 0.01 for $D_{A}^{{}^{\prime }}=1.0$, $D_{B}^{{}^{\prime }}=100.0$, $D_{C}^{{}^{\prime }}=0$ and *β* = 0.125, 0.5, 2.0, 8.0. For each set of parameters, we systematically simulate the dynamics for the selected sets of parameters and determine the output for spatial and temporal sensing. The strategy yielding the higher output will be selected. As shown in S1(a) and S1(b) Fig, *α* does not affect the choice of temporal and spatial sensing. We also plot the output using temporal sensing (green) versus spatial sensing (red) at *β* = 0.125 (S1c Fig) and *β* = 8.0 (S1d Fig) for different values of *α*. We observe that both the outputs scale linearly with the increase in *α*. Since *α* affects both outputs equally, it does not affect the sensing choice.

### High ratio of cell speed to cell diameter favors temporal sensing

**Fig 3:**
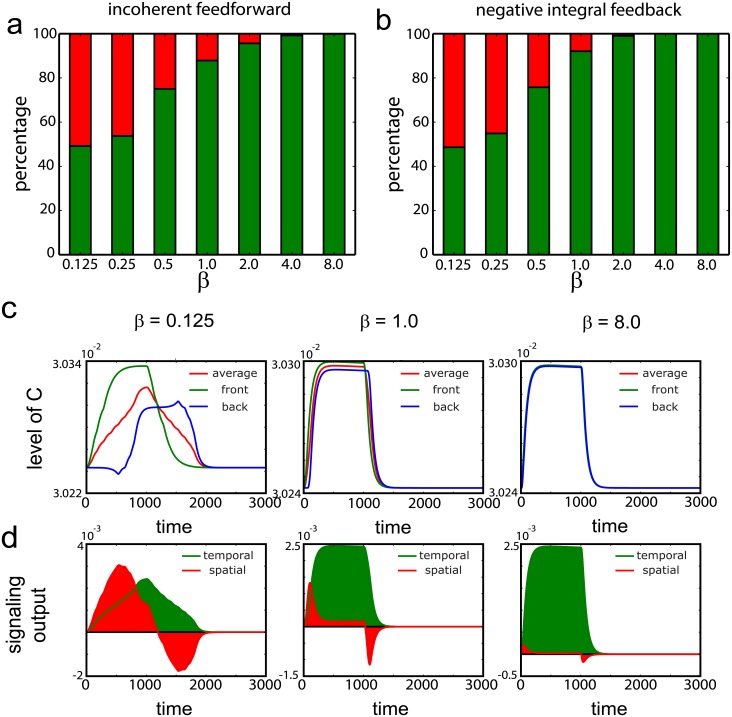
Temporal sensing is favored at high ratio of cell speed over cell size. Percentage of runs where temporal sensing yield high output (green) or spatial sensing yield high output (red) for (a) incoherent feedforward and (b) negative integral feedback circuits at different values of *β*. The range of $D_{A}^{{}^{\prime }}$ and $D_{B}^{{}^{\prime }}$ values used are $D_{A}^{{}^{\prime }}=0.1,1.0,10,100,1000$ and $D_{B}^{{}^{\prime }}=0.1,1.0,10,100,1000$. (c) Dynamics of the average level of protein *C* (red), level of protein *C* at the front (green) and back (blue) for different values of *β* for the incoheret feedforward circuit with $D_{A}^{{}^{\prime }}=D_{B}^{{}^{\prime }}=1.0$. (d) Output from temporal (green) and spatial sensing (red) for the different values of *β*.

Since *α* does not affect the sensing choice, we have fixed *α* = 0.001 and focus on the effects of $D_{A}^{{}^{\prime }}$, $D_{B}^{{}^{\prime }}$ and *β*. We simulate the protein dynamics for *β* = 0.125, 0.25, 0.5, 1.0, 2.0, 4.0, 8.0, $D_{A}^{{}^{\prime }}=0.1,1.0,100,1000$ and $D_{B}^{{}^{\prime }}=0.1,1.0,100,1000$. We plot the percentage of runs that yield higher signaling output adopting the temporal (green) and spatial (red) strategy for different values of *β* in Fig 3(a) and 3(b). Although the negative integral feedback (NFB) and incoherent feedforward circuits (IFF) have been associated with temporal [9, 10] and spatial sensing [11, 12] respectively, we find that the two circuits yield similar results. This shows that NFB can be used for spatial sensing and that the IFF can be used for temporal sensing. We find that when *β* is high (cell velocity is high or cell diameter is small), temporal sensing yields higher output than spatial sensing independent of the value of $D_{A}^{{}^{\prime }}$ and $D_{B}^{{}^{\prime }}$.

To examine the effect of *β*, we plot the protein dynamics of the output protein for one set of parameter for the incoherent feedforward circuit at various values of *beta* for $D_{A}^{{}^{\prime }}=1.0$ and $D_{B}^{{}^{\prime }}=1.0$. When *β* is small (cell velocity is small or cell diameter is large), the front and back halves of the cell experience a big delay in the time that they observe the chemoattractant and the levels of the output protein, *C*, at the rear end (blue curve) of the cell only increase after the level of *C* at the front end (green curve) starts to decrease (Fig 3c, left). The average level of *C* (red curve), which sums over the two halves, shows a net increase at all times when the cell is moving through the gradient. As *β* increases, the time difference in which the front and back halves of the cell experiences the chemoattractant decreases and their dynamics began to converge (Fig 3c, right).

The signaling output for temporal sensing (green) and spatial sensing (red) were plotted in Fig 3d. When the cell uses the temporal sensing mechanism by comparing the average output C with the baseline level, it observes a net increase in output (area shaded in green) as the cell moves through the gradient. When spatial sensing is used to comparing the ratio of output at the front and back of the cell, the cell observes an increase in output (area shaded in red above the x-axis) as the cell entered the gradient (entering phase) followed by a decrease in output (area shaded in red below the x-axis) as the cell exits the gradient (exit phase) (Fig 3d). However the area above the x-axis is always larger than the area below the x-axis indicating an overall positive response. As the difference between the level of the output protein at the front and back of the cell decreases with increasing *β*, so is the signal obtained from spatial sensing. This explains why at high *β*, temporal sensing is favored.

Rather than size cell, we show that the relevant parameter for sensing is the ratio of cell velocity to the product of signaling rate and cell diameter. This suggests that cells moving faster than its cell diameter in the time it takes for signaling to propagate across the cell diameter should adopt temporal sensing, whereas cells moving slower than its cell diameter in that time should adopt spatial sensing. This can be reasoned as follows: a fast-moving, small cell performs better comparing the chemoattractant at different times in its trajectory; whereas, a slow-moving, big cell that is not travelling much performs better by comparing the chemoattractant concentration at its two ends.

### Diffusivity of activator is smaller than diffusivity of repressor for spatial sensing

**Fig 4:**
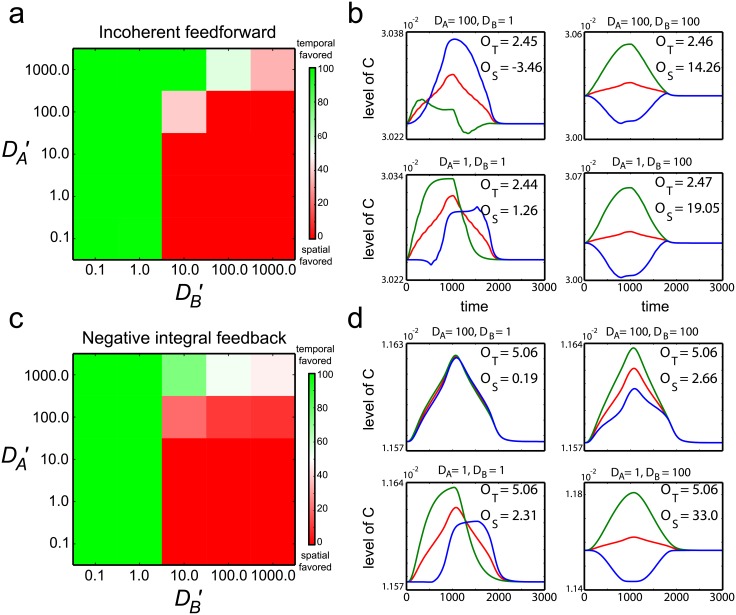
Temporal sensing is favored over spatial sensing when diffusion rate of activator is higher than that of the inactivator. (a,c) Fraction of parameters that choose temporal sensing over spatial sensing for different values of $D_{B}^{{}^{\prime }}$ and $D_{A}^{{}^{\prime }}$ at *β* = 0.125 for the incoherent feedforward (a) and negative integral feedback circuits (c). (b,d) Dynamics of the average level of protein *C* (red), level of protein *C* at the front (green) and back (blue) for different values of $D_{A}^{{}^{\prime }}$ and $D_{B}^{{}^{\prime }}$ for the incoherent feedforward (b) and negative integral feedback circuits (d). *O*_*T*_ and *O*_*S*_ are the output from temporal and spatial sensing respectively.

As shown in Fig 3a and 3b, both temporal and spatial sensing can occur when *β* is small and we will next focus on the effects of $D_{A}^{{}^{\prime }}$ and $D_{B}^{{}^{\prime }}$ on this sensing choice. As shown in Fig 4(a) and 4(c), temporal sensing is favored when $D_{A}^{{}^{\prime }}>D_{B}^{{}^{\prime }}$ and spatial sensing is favored when $D_{A}^{{}^{\prime }}<D_{B}^{{}^{\prime }}$. The dynamics of protein *C* are plotted for different values of $D_{A}^{{}^{\prime }}$ and $D_{B}^{{}^{\prime }}$ (Fig 4b and 4d). At low diffusion ($D_{A}^{{}^{\prime }}=1$ and $D_{B}^{{}^{\prime }}=1$), the front and back halves behave like separate uncommunicating entities as discussed before and temporal signaling yields slightly higher output than spatial sensing (Fig 4b and 4d, bottom row,left). When diffusion of the activator is slow and diffusion of the inactivator is fast ($D_{A}^{{}^{\prime }}=1$ and $D_{B}^{{}^{\prime }}=100$), coupling between the front and back of the cell occurred. Once the cell entered the gradient, inactivator *B* is produced and diffuses to the back of the cell to suppress the output level of protein *C*, amplifying the difference in levels of protein *C* between the front and the back. This amplification led to a reduction of *C* from its basal level at the back of the cell (Fig 4b and 4d, bottom row, right). Hence spatial sensing yields much higher output signal than temporal sensing. Furthermore, this coupling ensured that the levels of protein *C* at the back of the cell is lower than that at the front even during the exit phase. This is consistent with previous models adopting a local acting activator and a globally acting inactivator for spatial sensing [11].

On the other hand when diffusion of the activator is fast and diffusion of the inactivator is slow ($D_{A}^{{}^{\prime }}=100$ and $D_{B}^{{}^{\prime }}=1$), the global activation and local inhibition happens with activator diffusing to the back of the cell. In IFF, this leads to higher level of protein *C* at the back than the front during the entering phase (Fig 4b, top row, left). This occurs as protein *A* produces at the front end of the cell rapidly diffused to the back, homogenizing level of protein *A* throughout the cell. The higher level of inactivator, protein *B*, leads to greater repression and lower level of protein *C* at the front. In this case, the level of protein *C* becomes higher at the back and the signaling output from spatial sensing becomes negative, making spatial sensing an inviable option. This effect is not observed in NFB circuits as, protein *B* was activated by protein *A* rather than the external chemoattractant (Fig 4d, top row, left). Hence level of protein *B* is always be proportional to that of protein A. However in this case, spatial sensing is also not favored as the rapid diffusion of protein *A* led to loss of information about the external chemoattractant gradient. Finally when both activator and inactivator diffuse fast ($D_{A}^{{}^{\prime }}=100$ and $D_{B}^{{}^{\prime }}=100$), the amplification effect observed for local excitation and global inhibition is still observed, albeit at a lower value (Fig 4b and 4d, top row, right).

In summary, we find that spatial sensing is favored when the repressor diffuses faster than the activator. This is because repressor produces at the front end is able to diffuse to the back to lower the signaling level of the output protein. This magnifies the difference between the signal output at the two ends, leading to higher signaling output for spatial sensing. When repressor diffuses slower than the activator, this amplification does not occur and temporal sensing is favored.

To check that our findings are independent of the exact gradient profile, we repeat our analysis for an exponential gradient (S2 Fig). We find that similar to results of the linear gradient, high ratio of cell speed to cell diameter favors temporal sensing and diffusivity of activator has to be smaller than diffusivity of repressor for spatial sensing to be preferred at low values of *β*.

In our simulations, the cell is moving from a region of constant chemoattractant, into a region with a linear increase in chemoattractant and finally into another region of a higher constant chemoattractant level. In general, cells may be moving inside a steady state gradient. To show that the motion from a region of constant chemoattractant into a gradient does not affect the findings, we simulate the response of cells into a step change in chemoattractant (S3 Fig). This will simulate the case where a cell suddenly encounters a gradient and moves into it, as opposed to moving inside a steady-state gradient. We find that the main findings are consistent with those for a linear gradient. We also repeat the simulations using a longer *T*_*s*_ = 20 and obtain similar findings (S4 Fig).

### Ratio of cell speed to cell diameter determines sensing choices

**Fig 5:**
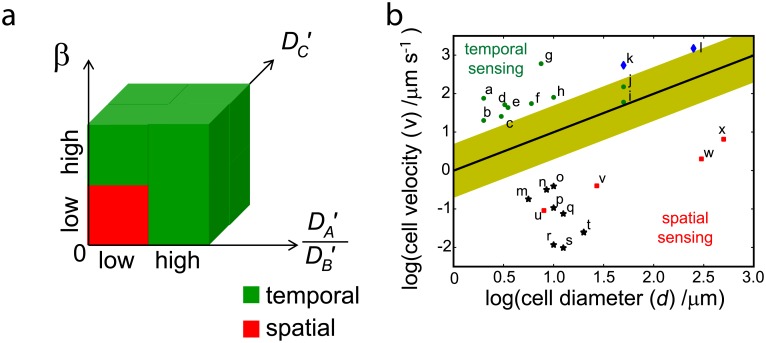
Choice of temporal versus spatial sensing. (a) Parameter space for choice of temporal versus spatial sensing. (b) Plot of log(cell diameter, *d*) versus log(cell velocity, *v*) for chemotactic cells. The respective cells are as follows; In the flagellar group (green dots) are a: *Vibriocholarae*, b: *Escherichia* *coli*, c: *Helicobacter* *pylori*, d: *Pseudomonas* *aeruginosa*, e: *Salmonella* *typhimurium*, f: Marine Vibrioid bacteria sampled from Niva Bay, g: *Thiovulum* *majus*, h: *Chlamydomonas* *reinhardtii*, i: Sperm (human) and j: Sperm (Sea urchin). In the cilia group (blue diamonds) are k: *Tetrahymena* *thermophila* and l: *Paramecium*. In the lamellipodia/filopodia group (black stars) are m: T cell, n: Neutrophil, o: Hemocyte, p: B cell, q: Dendritic cell, r: Endothelial cell, s: Fibroblast and t: Border cells. In the pseudpodia group (red squares) are u: *Dictyostelium* *discoideum*, v: *Acanthamoeba* *castellanii*, w: *Amoeba* *proteus* and x: *Chaos* *carolinensis*. Assuming a signaling rate, *l*_*BC*_, occurring between 0.2*s*^−1^ to 5*s*^−1^ then the yellow region will be the separating boundary between cells with high and low values of *β*. The black line is the decision boundary for *l*_*BC*_ = 1*s*^−1^.

In Fig 5a, we summarize our findings. We find that sensing outcomes are determined by three dimensionless parameters: 1) the ratio of cell speed to the product of cell diameter and rate of signaling, 2) the diffusivities of the output protein of the two circuits and 3) the ratio of the diffusivities of the activator to inactivator protein. Temporal sensing is usually preferred whereas spatial sensing is preferred when all three parameters are low.

To compare our theoretical results with experimental observations, we need to determine the diffusion rates, cell sizes and speeds of a wide range of chemotactic cells and organisms. While cell sizes and speeds are readily available, values of diffusion rates are much harder to find. Hence, we first compare our findings based on the ratio of cell speed to cell diameter with that of the sensing decisions of chemotactic cells and organisms. The most well-studied chemotactic organism is *E*. *coli*. *E*. *coli* is 2 *μm* in length [15] and swims at about 20 *μm*/*s*. The dephosphorylation rate of Che-Y has been found to be 2.2*s*^−1^[16, 17]. This yields *β* = 4.5, agreeing with our analysis that *E*. *coli* will adopt temporal sensing. Since reactions rates are difficult to characterize and the circuitry controlling chemotaxis is usually much more complicated than our canonical NFB and IFF circuits, we are unable to obtain *l*_*BC*_ for many chemotactic cells. Nonetheless, we estimate reaction rates to be of the order of seconds based on the dephosphorylation rate of Che-Y [16, 17] and the fast response time observed in chemotactic cells. Micropipette stimulation experiments showed that neutrophils took between 5–30*s* to extend their surface towards the chemotactic pipette [18].

We conduct an extensive literature search to obtain the diameters and velocities of many chemotactic cells and unicellular organisms such as bacteria [19–22], *Paramecium* *caudatum* (*P*. *caudatum*) [23], *Tetrahymena* *thermophila*[24], alga [25]; sperm cells [26, 27]; mammalian cells [28–33]; insect cells [34, 35]; and amoeba [36–38]. We classified these chemotactic cells based on their mechanisms of motion, namely lamellipodia/filopodia, flagellar, pseudpodia and cilia. In general, the eukaryotic and insect cells are in the lamellipodia/filopodia group; bacteria and sperm cells are in the flagellar group; amoeba are in the pseudpodia group; and *Tetrahymena* *thermophila* and alga are in the cilia group. We find that cells using flagellar and cilia to move have higher ratio of velocity over cell diameter than cells using lamellipodia/filopodia and pseudpodia (Fig 5b). In our simulations, we find that cells and organisms with high ratio of cell speed to cell diameter adopt temporal sensing. Assuming *l*_*BC*_ = 1*s*^−1^, cells and organisms above the the black horizontal line in Fig 5b will adopt temporal sensing. In general, *l*_*BC*_ may be different in each cell, if *l*_*BC*_ lies between 0.2*s*^−1^ − 5*s*^−1^ then the yellow region will be the separating boundary between cells with high and low values of *β*. Cells with high *β* values includes cells in the flagellar (green) group and agrees with the broad categorization that these cells adopt temporal sensing. Sperm cells have been shown to utilize temporal sensing despite being relatively big [26]. Our results suggests that temporal sensing is utilized as its high cell velocity makes temporal sensing more advantageous. One exception to the classification is the bipolar flagellated vibrioid bacteria that has been suggested to adopt spatial sensing [39]. This bacteria has a very fast response time as it was able to correct deviations from its swimming direction within a second. Further work elucidating the chemotaxis circuitry and reaction rates in this organism is necessary to determine the value of *β*. It is currently unclear whether *P*. *caudatum* adopts spatial or temporal sensing. The other ciliated organism (blue), *Tetrahymena* *thermophila*, has been proposed to utilize temporal sensing [40], agreeing with our prediction.

Cells and organisms below the yellow region in Fig 5b have low values of *β*. We find that these cells would adopt spatial sensing if the activator diffuses slowly whereas the inactivator diffuses fast. Unfortunately it is difficult to obtain these diffusion rates as many of the activator and inactivator proteins involved are unknown. For example, in *Dictyostelium* *discoideum*, some literature suggests that the locally acting activator (Protein *A*), PI3-kinase, and globally acting inactivator (Protein *B*), PTEN, work together to control G-protein (Protein *C*) activation during chemotaxis [13] whereas other literature suggests that RasGEF and a RasGAP are the activator and inactivator proteins instead [12]. As diffusion rate is inversely proportional to the square root of the molecular weight, one could estimate the ratio of PTEN to PI3K diffusion rate and the ratio of RasGEF to RasGAP diffusion rate to be $\sqrt[{2}]{\frac{83,598}{47,166}}=1.33$ and $\sqrt[{2}]{\frac{57,010}{54,556}}=1.02$ respectively. The slight differences in these estimated diffusion rates are clearly inconsistent with the local and global activation roles suggested. This shows that even when there are candidate proteins for the activator and inactivator proteins, molecular weight is not a good approach for estimating diffusion rates in cells and suggests the presence of other active biological processes in controlling the movements of these proteins.

From Fig 4(b) and 4(d), we observe that the signaling output, *O*_*S*_, is highest at low activator diffusion rate and high inactivator diffusion rate ($D_{A}^{{}^{\prime }}=1.0$ and $D_{B}^{{}^{\prime }}=100$) for low value of *β*. From an evolutionary point of view, this suggests that organisms would evolve towards having high $D_{B}^{{}^{\prime }}$ and low $D_{A}^{{}^{\prime }}$ to achieve better chemotactic response. Indeed, it has been shown experimentally that lamellipodia/filopodia (black) and pseudpodia cells (red) utilize spatial sensing. Hence we find that *β* is the most important determinant in the choice between spatial and temporal sensing.

### Noise in the external chemoattractant favors temporal sensing

Next, we consider the effect of noise on the decision choice. Noise can exist in both the external chemoattractant and the internal signaling pathway and affects chemotaxis [41]. We focus our analysis on the regime where $D_{A}^{{}^{\prime }}$ is low and $D_{B}^{{}^{\prime }}$ is high as this was the region of parameter space that yields most interesting behavior in the deterministic analysis. We examine the decision choice for the following cases: (1) *β* = 0.25, (2) *β* = 1.0 and (3) *β* = 4.0 at $D_{A}^{{}^{\prime }}=1$ and $D_{B}^{{}^{\prime }}=100$ as the amount of external or internal noise increases. Since each run is stochastic, ten runs are performed on each set of parameters and noise level to determine the average performance from spatial and temporal sensing.

First, we focus on the presence of external noise in the chemoattractant gradient. The dynamics of protein *C* is plotted at different noise levels for *β* = 0.25 and *β* = 4.0 (S5 Fig). *η* quantifies the amount of fractional noise. At low level of noise, *η* = 0.0625, the dynamics of protein *C* is well behaved with the levels of protein *C* at the front always higher than that at the back (S5a and S5d Fig). As *η* increases, the dynamics becomes noisier with levels of protein *C* showing more fluctuations (S5b and S5e Fig). Furthermore, the level of protein *C* at the front of the cell is sometimes lower than that at the back. However, the average levels of protein *C* is still rather well-behaved, rising as the cell enters the chemoattractant gradient and adapting back to basal level as the cell exits the gradient. At high level of noise, *η* = 1.0, the noise level dominates over the signal and the levels of protein *C* fluctuated randomly (S5c and S5f Fig).

**Fig 6:**
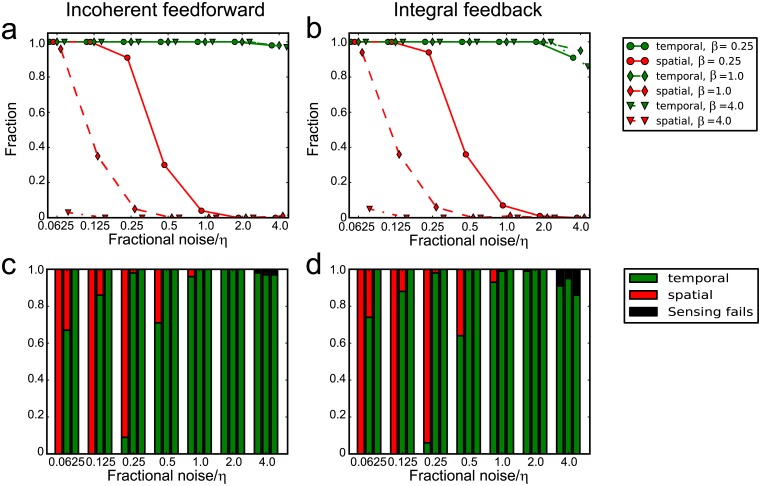
Noise in the external chemoattractant favors temporal sensing. Results for the incoherent feedforward circuit (left) and negative integral feedback circuit (right). (a) Fraction of parameters that yielded *O*_*T*_ > 0 (green) and *O*_*S*_ > 0 (red) for all the stochastics runs at different amount of noise, *η*, for *β* = 0.25 (circle), *β* = 1.0 (diamonds) and *β* = 4.0 (triangles). (b) Fraction of parameters that chose temporal sensing (green), spatial sensing (red) and failure in sensing (black) at different amount of noise for *β* = 0.25 (first column), *β* = 1.0 (second column) and *β* = 4.0 (third column).

Next, we want to determine which sensing strategy is more susceptible to noise. For each set of parameters, ten stochastic runs are performed. If all the runs yield positive signaling output for a particular sensing strategy that strategy is considered to be viable for that set of parameters. We plot the fraction of parameter set that fulfil the above criteria for spatial and temporal sensing (Fig 6a and 6b (red). We find that temporal sensing (green) was less susceptible to noise than spatial sensing (red). Intuitively, this can be understand as taking average in temporal sensing is more robust than taking difference in spatial sensing. We also find that the fraction of parameters fulfilling the criteria increased as *β* decreased (Fig 6a, (red)). This showed that spatial sensing is less susceptible to noise when the cell diameter is larger than cell velocity.

Lastly, we determine the fraction of parameters that chooses temporal or spatial sensing. When noise level becomes too high, both sensing mechanisms fail as the signal had been completely dominated by noise. As shown in (Fig 6c and 6d), spatial sensing performs better than temporal sensing for low values of *β* and low values of noise. As noise level increases, temporal sensing yields better results. Finally at very high noise levels, sensing using both strategies are infeasible.

### Noise in the internal signaling pathway does not affect sensing choice

**Fig 7:**
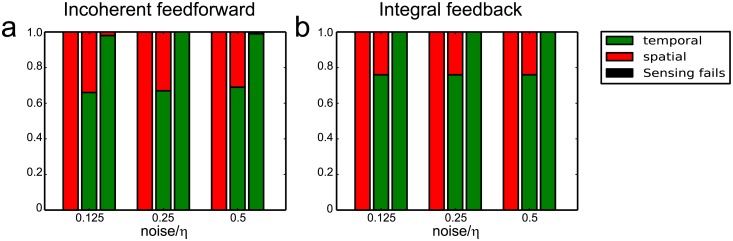
Internal noise does not affect sensing choice. Fraction of parameters that chose temporal sensing (green), spatial sensing (red) and failure in sensing (black) at different amount of noise, *ν*, for *β* = 0.25 (first column), *β* = 1.0 (second column) and *β* = 4.0 (third column) for incoherent feedforward circuit (a) and negative integral feedback circuit (b).

**Fig 8:**
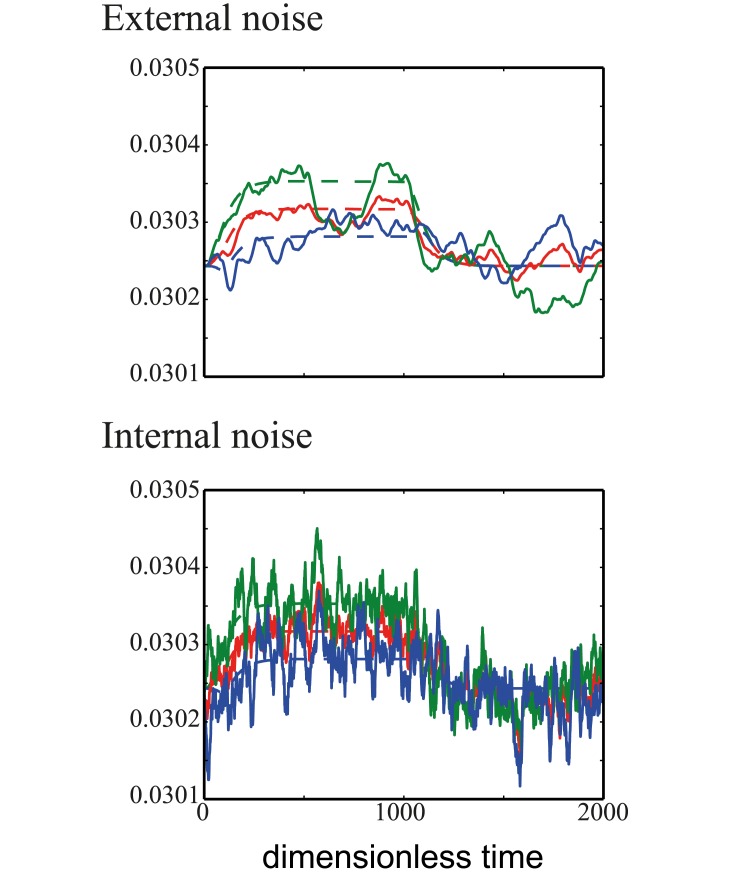
Dynamics of the average level of protein *C* (red), level of protein *C* at the front (green) and back (blue) for noise in the external chemoattractant gradient (top) and internal signaling pathway (bottom). Dotted lines are the average level of protein in the absence of any noise. Values used are *β* = 1.0, $D_{A}^{{}^{\prime }}=1.0$, $D_{B}^{{}^{\prime }}=100.0$ and *η* = 1.0 (top) and *ν* = 0.25 (below).

To introduce noise into the internal signaing pathway, we allow all the kinetic parameters (*k*_*IA*_, *k*_*IB*_, *l*_*FA*_, *l*_*FB*_, *k*_*AC*_, *l*_*BC*_, *k*_*CB*_) to be random variable with mean equal to their values in the noiseless case and variance, *ν*. We find that the sensing decision is independent of the amount of noise, *ν* (Fig 7). We examine the dynamics of protein *C* when subjected to external chemoattractant noise and internal signaling noise (Fig 8). We find that in the presence of internal noise, protein *C* fluctuates at high frequency about the expected value of *C* for the noiseless case. Integrating over time, the noise would cancel out, leading to an average performance similar to that of the noiseless case. On the other hand, protein *C* fluctuates at low frequency in the presence of external noise and its mean averaged over time can be quite different from the expected value of *C* for the noiseless case. Hence in this case, temporal sensing performs better.

## Discussion

Here, we determine the conditions favoring temporal and spatial sensing. We find that the behavior of the negative integral feedback and incoherent feedforward circuits were determined by five dimensionless constants, namely the three diffusion rates, $D_{A}^{{}^{\prime }}=\frac{D_{A}}{d^{2}l_{BC}}$, $D_{B}^{{}^{\prime }}=\frac{D_{B}}{d^{2}l_{BC}}$ and $D_{C}^{{}^{\prime }}=\frac{D_{C}}{d^{2}l_{BC}}$, the ratio of cell speed to the product of cell diameter and signaling rate, $\beta =\frac{v}{dl_{BC}}$, and the effective chemoattractant gradient, $\alpha =\frac{vk}{l_{BC}}$. Both the negative integral feedback and incoherent feedforward circuits yielded similar behaviors when we varied the dimensionless constants. We summarize our findings in Fig 5a. In brief, temporal sensing is favored in most situations whereas spatial sensing is only favored when values for $D_{C}^{{}^{\prime }}$ and *β* are small and $D_{A}^{{}^{\prime }}$, diffusion rate of the activator, is slower than that of $D_{B}^{{}^{\prime }}$, diffusion rate of the inactivator. Comparing our findings with experimental observations, we found that the sensing choices observed experimentally agrees with that predicted based on their values of *β*. This does not mean that the other requirements on the diffusion rates are not important. We speculate that when *β* is low, the proteins would evolve over time to fulfil the requirements for spatial sensing as this strategy can yield much higher output than temporal sensing.

We use the simplest form of NFB and IFF circuitries to study chemotaxis as they capture the requirements of perfect adaptation and senstivity in chemotaxis. Previous mathematical models put forward to explain spatial sensing [11, 13, 42–44] are more complicated versions of these circuits. Levchenko’s model is similar to the IFF circuit [13]; Hans’ model is an IFF circuit with two inhibitors [42]; and Ma’s model consists of two competing IFF circuits acting in parallel [11]. Narang’s and Rappel’s models are reminiscent of the NFB loop as simulation of an activator leads to production of a second messanger that inhibits the activator [43, 44]. Through our simulations, we find that $D_{A}^{{}^{\prime }}<D_{B}^{{}^{\prime }}$ is required for spatial sensing to be favored over temporal sensing. Indeed, this is also an assumption found in the previous models [11, 13, 42–44]. For example, membrane-bound PI4,5P_2_ with low diffusivity and cytosol IP_3_ with high diffusivity are proposed to be the activator and inhibitor respectively in Narang’s model [44]; whereas locally acting PI3-kinase and globally acting inactivator PTEN are suggested to be the activator and inhibitor respectively in Levchenko’s model [13]. There have also been recent works that modify the NFB and IFF circuitries to account for other behaviours observed in chemotaxis like fold-change detection of the chemoattractant [45] and rectified directional sensing [46]. Nonetheless, our simple circuits are able to reproduce the conditions necessary in other more complicated mathematical models and serve as good starting points for analyzing the impact of the different parameters.

NFB circuit is often associated with temporal sensing [9, 10] whereas IFF circuit is associated with spatial sensing [11, 13]. This is in part historical due to the success of the NFB circuit in explaining temporal sensing for E. coli and IFF circuit in explaining spatial sensing for social amoeba. Recently, it was found that the IFF circuit, but not the NFB circuit, can explain the faster protein adaptation dynamics in social amoeba at higher chemoattractant stimulation [12]. This is a clear experimental result supporting the use of IFF circuit for spatial sensing while ruling out the NFB circuit. Here, we found that both the circuits yielded similar results and could be use for both temporal and spatial sensing. It remains to be seen whether the NFB circuit offers general advantages over IFF circuit for temporal sensing and vice versa. To answer this question, one needs to find inputs where the two circuits behave differently. Indeed, it was found that when subjected to a ramp input, IFF circuit adapt perfectly whereas NFB circuit come to a steady-state activity proportional to the gradient of the ramp [47]. It is argued that temporal sensing, which involves sampling concentrations in space, is measuring the rate of change of the input. This makes the NFB suited for temporal sensing as its steady state response is proportional to the gradient of the ramp [48].

Incorporating noise into the external chemoattractant, we found that spatial sensing is more suspectible to noise than temporal sensing. This suggests that temporal sensing which averages over the signal across the entire cell is more robust than spatial sensing which takes the difference between the front and back of the cell. However, we note that when for low *β*, spatial sensing still performs better than temporal sensing for low noise level. This may be parameter regime where cells adopting spatial sensing operates in. Strategies like receptor coupling [49], memory [50] and cell-cell communication [51] could also be used in combination with our basic negative integral feedback and incoherent feedforward circuitries to buffer effects of noise and improve performance of spatial sensing.

## Materials and methods

### Modeling of chemotaxis

#### Step 1: Setting up equations for the negative integral and incoherent feedforward circuits

The negative integral (NFB) and incoherent feedforward (IFF) circuits have been found to be able to achieve adaptation, an important property required for cells to response to a wide range of chemoattractant concentration [14]. The equations for the NFB and IFF are as follows:

$$\begin{array}{cc} \frac{dA}{dt} & =k_{IA}I-l_{FA}A, \end{array}$$ (1a)

$$\begin{array}{cc} \frac{dB}{dt} & =\{\begin{array}{c} k_{CB}C-l_{FB},\;\;\;\;\;\;\;\text{(NFB),}\;\text{or}\\ k_{IB}I-l_{FB}B,\;\;\;\;\;\;\text{(IFF)} \end{array} \end{array}$$ (1b)

$$\begin{array}{cc} \frac{dC}{dt} & =k_{AC}A\frac{1-C}{K_{AC}+1-C}-l_{BC}B\frac{C}{L_{BC}+C}, \end{array}$$ (1c)

where *k*_*IA*_, *k*_*IB*_, *k*_*AC*_, *l*_*FA*_, *l*_*FB*_, *l*_*BC*_, *K*_*AC*_ and *L*_*BC*_ are the rate and equilibrium constants. The species in the equations represent the activated forms of the proteins.

These circuits consist of three proteins, the activator (*A*), the inactivator (*B*) and output protein (*C*) (Fig 2, step 1). The role of the activator (*A*) is to transduce the gradient input into the circuit whereas the output protein (*C*) serves as the readout. The inactivator (*B*) is required for achieving adaptation and it performs this role differently for the two circuits. In NFB, *B* is activated by *C* hence it monitors the level of *C* directly and feedback to adjust the level of *C* to the input-independent steady-state value [14]. In IFF, *B* is activated by the input and acts oppositely as *A* on *C* to reduce its activation. Although *B* does not actively monitor the level of *C*, it can anticipate *C*’s level and dampen it optimally as *B* is activated propoprtionally to *A*.

Eq (1) represents the most general form of the IFF and NFB circuits. We make them dimensionless and also adapt them for gradient sensing. These modifications are found in S1 Text.

#### Step 2: Identification of parameters that yield high sensitivity and precision

While the equations of the two circuits (Eq 1) are well-defined from step 1, the parameters for the rate and equilibrium constants are not and in general can take on many different values. Furthermore, certain choice of parameters may not yield adaptative response within our simulation time-scale. Hence we determine appropriate values for the rate and equilibrium constants by sampling 1,000,000 parameter sets to obtain parameters that achieve adaptation [14, 52]. For each parameter set, we solve for the dynamics of a cell represented as a single point after it experiences a step increase in signal at time *τ* = 0, which is the dimensionless time (Fig 2, step 2, left).

The performance of the circuits are quantified by their sensitivity, S, and precision, P, as defined below

$$\begin{array}{cc} S & =\frac{\left(C_{max}-C_{initial}\right)/C_{initial}}{\left(I_{H}-I_{L}\right)/I_{L}}, \end{array}$$ (2a)

$$\begin{array}{cc} P & =\frac{C_{initial}/\left(C_{final}-C_{initial}\right)}{I_{L}/\left(I_{H}-I_{L}\right)}, \end{array}$$ (2b)

where *C*_*initial*_, *C*_*max*_ and *C*_*final*_ are the initial concentration, maximum concentration and final concentration of *C* during the simulation time, *I*_*L*_ is the initial chemoattractant concentration and *I*_*H*_ is the final step input chemoattractant concentration. Sensitivity and precision are normalized with respect to the change in chemoattractant to capture the system’s response to the chemoattractant inputs.

High sensitivity is responsible for signal amplification in shallow gradients whereas high adaptation precision is required for signal adaptation that allows the cell to remain responsive to a high range of chemoattractant concentration (Fig 2, step 2, right). We selected 100 sets of parameters yielding high sensitivity and precision for further analysis. Please refer to the S1 Text for more details on how the parameters are obtained.

#### Step 3: Simulation of protein dynamics as cell moves through a gradient

Next, we model the dynamics of the proteins in the two circuits as the cell passes through a linear gradient using the selected parameter sets. A cell is modeled as a one-dimensional circular system with diameter, *d*, to capture the distribution of the proteins on the cell membrane (Fig 2, step 3). The proteins, *A*, *B* and *C*, are also allowed to diffuse on the cellular membrane with diffusion constants, *D*_*A*_, *D*_*B*_ and *D*_*C*_, respectively. Initially, the cell experiences chemoattractant input of *I* = *I*_*L*_. At time *τ* = 0, the cell moves at velocity, *v*, into a chemical gradient with slope, *k*, for a fixed time *T*_*s*_ before moving back into a region with constant *I* = *I*_*H*_ (Fig 2, step 3).

#### Step 4: Quantify temporal and spatial output

After passing through the linear gradient, the cell integrates the outputs for both the NFB and IFF circuits using temporal and spatial sensing to determine which sensing method yielded higher total output. In temporal sensing, the cell compares the level of *C* when it is passing through the linear gradient with the steady state level of *C* (area highlighted in green) (Fig 2, step 4, temporal). In spatial sensing, the cell compares the level of *C* at the front half and back half of the cell (area highlighted in red) (Fig 2, step 4, spatial).

Mathematically, the total output signal for the temporal sensing, *O*_*T*_, and spatial sensing, *O*_*S*_, are described as:

$$\begin{array}{cc} O_{T} & =\frac{\int_{0}^{\tau }\sum_{i=1}^{N}\left(\frac{C_{i}\left(\tau \right)}{C_{i}\left(0\right)}-1\right)d\tau }{N}, \end{array}$$ (3a)

$$\begin{array}{cc} O_{S} & =\int_{0}^{\tau }\frac{\sum_{i\in front}C_{i}\left(\tau \right)}{\sum_{i\in back}C_{i}\left(\tau \right)}-1d\tau . \end{array}$$ (3b)

To determine which sensing mechanism is better, we calculate the ratio,

$$\begin{array}{c} O_{TS}=\frac{O_{T}}{O_{S}}. \end{array}$$ (4)

When *O*_*TS*_ > 1, temporal sensing is favored. Otherwise, spatial sensing is favored. More details of the model can be found in S1 t.

## Supporting information

S1 Text Supplementary methods with a detailed description on (1) identification of parameter sets that yield high sensitivty and precision, (2) simulation of cell dynamics in chemoattractant concentration gradient, (3) modelling noise in the external chemoattractant, (4) modelling noise in the internal signaling pathway and (5) modelling other external chemoattractant profile. (PDF)

S1 Fig*α* does not affect the choice of temporal and spatial sensing.Fraction of parameters that choose temporal sensing over spatial sensing for various values of *α* and *β* for the (a) incoherent feedforward circuit and (b) integral feedback circuits. Values for $D_{A}^{{}^{\prime }}$ and $D_{B}^{{}^{\prime }}$ are 1.0 and 100.0 respectively. Temporal (green) and spatial (red) outputs for (c) *β* = 0.125 and (d) *β* = 8.0 as *α* changes for the incoherent feedforward circuit.(EPS)

S2 FigTemporal and spatial sensing choices in exponential gradient.(a,b) Percentage of runs where temporal sensing yield high output (green) or spatial sensing yield high output (red) for (a) incoherent feedforward and (b) negative integral feedback circuits at different values of *β*. The range of $D_{A}^{{}^{\prime }}$ and $D_{B}^{{}^{\prime }}$ values used are $D_{A}^{{}^{\prime }}=0.1,1.0,10,100$ and $D_{B}^{{}^{\prime }}=0.1,1.0,10,100$. (c,d) Fraction of parameters that choose temporal sensing over spatial sensing for different values of $D_{B}^{{}^{\prime }}$ and $D_{A}^{{}^{\prime }}$ at *β* = 0.25 for the incoherent feedforward (c) and negative integral feedback circuits (d).(EPS)

S3 FigTemporal and spatial sensing choices when experiencing a step change in concentration.(a,b) Percentage of runs where temporal sensing yield high output (green) or spatial sensing yield high output (red) for (a) incoherent feedforward and (b) negative integral feedback circuits at different values of *β*. The range of $D_{A}^{{}^{\prime }}$ and $D_{B}^{{}^{\prime }}$ values used are $D_{A}^{{}^{\prime }}=1.0,10,100$ and $D_{B}^{{}^{\prime }}=1.0,10,100$. (c,d) Fraction of parameters that choose temporal sensing over spatial sensing for different values of $D_{B}^{{}^{\prime }}$ and $D_{A}^{{}^{\prime }}$ at *β* = 0.25 for the incoherent feedforward (c) and negative integral feedback circuits (d).(EPS)

S4 FigTemporal and spatial sensing choices for a linear gradient of longer duration.(a,b) Percentage of runs where temporal sensing yield high output (green) or spatial sensing yield high output (red) for (a) incoherent feedforward and (b) negative integral feedback circuits at different values of *β* for *T*_*S*_ = 20. The range of $D_{A}^{{}^{\prime }}$ and $D_{B}^{{}^{\prime }}$ values used are $D_{A}^{{}^{\prime }}=1.0,10,100$ and $D_{B}^{{}^{\prime }}=1.0,10,100$. (c,d) Fraction of parameters that choose temporal sensing over spatial sensing for different values of $D_{B}^{{}^{\prime }}$ and $D_{A}^{{}^{\prime }}$ at *β* = 0.25 for the incoherent feedforward (c) and negative integral feedback circuits (d).(EPS)

S5 FigNoise in signaling output increases with noise in the external chemoattractant.Dynamics of the average level of protein *C* (red), level of protein *C* at the front (green) and back (blue) for different values of *β* and *η* for $D_{A}^{{}^{\prime }}=1.0$ and $D_{B}^{{}^{\prime }}=100.0$.(EPS)
