# Supplementary material for: A computational model for how cells choose temporal or spatial sensing during chemotaxis
Source: PLoS Comput Biol. 2018 Mar 5;14(3):e1005966. doi: 10.1371/journal.pcbi.1005966 (PMC5854446; doi:10.1371/journal.pcbi.1005966)
Supplement: S1 Text — (PDF) [file pcbi.1005966.s001.pdf]

# Supplementary methods

Our simulation to determine the performance of temporal and spatial sensing consists of three parts; (i) identification of parameters sets for the integral feedback and incoherent feedforward circuits that yield high sensitivity (high output response to the input) and adaptation precision (difference between pre and post-stimulus levels), (ii) simulations to obtain the dynamics for cell moving in a chemoattractant gradient and (iii) signal output comparisons between temporal and spatial sensing.

## Identification of parameter sets that yield high sensitivty and precision

First, we identified parameters for the negative integral feedback (NFB) and incoherent feedforward (IFF) circuits that yielded high sensitivity and adaptation precision by subjecting the cells to a step increase in chemoattractant level. High sensitivity and adaptation precision were two properties that were required for gradient sensing; high sensitivity was responsible for signal amplification in shallow gradients whereas high adaptation precision was required for signal adaptation that allows the cell to remain responsive to a high range of chemoattractant concentration. At this point, a cell could be thought of as a single point and its proteins were homogenous and constant across the cell.

The equations describing the dynamics for the activator (protein $A$) and the output protein (protein $C$) were similar for the two circuits. The equations for the inactivator (protein $B$) were different. The equations for the incoherent feedforward and integral feedback were as follows:

$$\frac{dA}{dt} = k_{IA}I - l_{FA}A, \tag{1a}$$

$$\frac{dB}{dt} = \begin{cases} k_{IB}I\frac{1-B}{(1-B)+K_{IB}} - l_{FB}B\frac{B}{B+L_{FB}}, & \text{(IFF), or} \\ k_{CB}C\frac{1-B}{(1-B)+K_{CB}} - l_{FB}\frac{B}{B+L_{FB}}, & \text{(NFB)} \end{cases} \tag{1b}$$

$$\frac{dC}{dt} = k_{AC}A\frac{1-C}{K_{AC}+1-C} - l_{BC}B\frac{C}{L_{BC}+C}, \tag{1c}$$

where $k_{IA}$, $k_{IB}$, $k_{AC}$, $l_{FA}$ ,$l_{FB}$ ,$l_{BC}$, $K_{CB}$, $K_{IB}$, $K_{AC}$, $L_{FB}$ and $L_{BC}$ were the rate and equilibrium constants. In both circuits, protein $A$ was the activator whereas protein $B$ wais the repressor as they activated and deactivated protein $C$, the output protein, respectively. Although protein B served as the repressor in both circuits, there were some differences in the two

circuits. In NFB, protein $B$ was activated by protein $C$, whereas, in the IFF, protein $B$ was activated by the external signal. In NFB, it has been shown that perfect adaptation is achieved when the enzymes acting on $B$ is in the saturating regime ($(1-B) >> K_{CB}$ and $B >> L_{FB}$). Similarly in IFF, perfect adaptation is achieved when the first term and second term are in the saturated regime and linear regime respectively ($(1-B) >> K_{AB}$ and $B << L_{FB}$).

With the above assumptions, the equations for the incoherent feedforward and integral feedback can be simplified into:

$$\frac{dA}{dt} = k_{IA}I - l_{FA}A, \tag{2a}$$

$$\frac{dB}{dt} = \begin{cases} k_{IB}I - l_{FB}B, & \text{(IFF), or} \\ k_{CB}C - l_{FB}, & \text{(NFB)} \end{cases} \tag{2b}$$

$$\frac{dC}{dt} = k_{AC}A\frac{1-C}{K_{AC}+1-C} - l_{BC}B\frac{C}{L_{BC}+C}, \tag{2c}$$

where $k_{IA}$, $k_{IB}$, $k_{AC}$, $l_{FA}$ ,$l_{FB}$ ,$l_{BC}$, $K_{AC}$ and $L_{BC}$ were the rate and equilibrium constants.

To make eqns. (2) dimensionless, we set $\tau = l_{BC}t$ and obtained

$$\frac{dA}{d\tau} = k'_{IA}I - l'_{FA}A, \tag{3a}$$

$$\frac{dB}{d\tau} = \begin{cases} k'_{IB}I - l'_{FB}B, & \text{(IFF), or} \\ k'_{CB}C - l'_{FB}, & \text{(NFB)} \end{cases} \tag{3b}$$

$$\frac{dC}{d\tau} = k'_{AC}A\frac{1-C}{K_{AC}+1-C} - B\frac{C}{L_{BC}+C}, \tag{3c}$$

where $k'_{IA}$, $k'_{IB}$, $k'_{AC}$, $l'_{FA}$ ,$l'_{FB}$ , $K'_{AC}$ and $L'_{BC}$ were the rate and equilibrium constants after normalizing with $l_{BC}$.

We quantify the cell's response to the chemoattractant by its sensitivity, $S$, and precision, $P$, as defined below

$$S = \frac{(C_{max} - C_{initial})/C_{initial}}{(I_H - I_L)/I_L}, \tag{4a}$$

$$P = \frac{C_{initial}/(C_{final} - C_{initial})}{I_L/(I_H - I_L)}, \tag{4b}$$

where $C_{initial}$, $C_{max}$ and $C_{final}$ are the initial concentration, maximum concentration and final concentration of $C$ during the simulation time, $I_L$ is the initial chemoattractant concentration and $I_H$ is the final step input chemoattractant concentration. Sensitivity and precision are normalized with respect to the change in chemoattractant to capture the system's response to the chemoattractant inputs.

To select parameters that yield high sensitivity and precision, we obtain 1,000,000 parameter sets by random sampling in logarithmic scale for both network topologies. We simulate the dynamics for the two circuits, sampling parameters from the following range: $10^{-2} < k_{IA}, k_{IB}, k_{AC}, l_{FA}, l_{FB} < 10^2$ and $10^{-3} < K_{AC}, L_{BC} < 10^2$. Values for the other parameters are $\tau = 5$, $I_L = 0.5$ and $I_H = 0.6$. After each run, the sensitivity and precision achieved for each set of parameters are determined using eqns. (4). Finally from the set of parameters that yield high sensitivity ($S > 1$) and precision ($P > 10$), we select parameters that lead to increase in $C$ to yield circuits that react in a positive manner to the chemoatractant. From this selected set, we randomly select 100 sets of parameters for further analysis.

## Simulation of cell dynamics in chemoattractant concentration gradient

In the second part, we model the dynamics of the various proteins as the cell passes through a linear gradient using the selected parameter sets obtained from the first part. After passing through the linear gradient, the cell integrates the outputs for both the integral feedback and incoherent feedforward circuits using temporal and spatial sensing to determine which sensing method yields higher total output.

For comparison of temporal versus spatial sensing, we extend eqns.(2) and model the cell as a one-dimensional circular system with diameter, $d$, to capture the distribution of the proteins on the cell membrane (Fig. **??**d, left). Furthermore, the proteins can diffuse on the cellular membrane. The diffusion constants for proteins, $A$, $B$ and $C$ were $D_A$, $D_B$ and $D_C$ respectively. Initially, the cell experiences chemoattractant input of $I = I_L$. At time $\tau = 0$, the cell moves at velocity, $v$, into a chemical gradient with slope, $k$, for a fixed time $T_s$ before moving back into a region with constant $I = I_H$. Extending the integral feedback and incoherent feedforward equations to allow for diffusion on a circular membrane, we obtain the following dimensionless equations:

$$\frac{dA_i}{d\tau} = k'_{IA}I - l'_{FA}A + D'_A(A_{i+1} + A_{i-1} - 2A_i), \tag{5a}$$

$$\frac{dB_i}{d\tau} = \begin{cases} k'_{IB}I - l'_{FB}B + D'_B(B_{i+1} + B_{i-1} - 2B_i), & \text{(IFF), or} \\ k'_{CB}C - l'_{FB} + D'_B(B_{i+1} + B_{i-1} - 2B_i), & \text{(NFB)} \end{cases} \tag{5b}$$

$$\frac{dC_i}{d\tau} = k'_{AC}A\frac{1-C}{K_{AC}+1-C} - B\frac{C}{L_{BC}+C} + D'_C(C_{i+1} + C_{i-1} - 2C_i), \tag{5c}$$

$$\frac{dI_i}{d\tau} = \alpha\frac{sign(\frac{1}{2}cos(id\theta) - \frac{1}{2} + \beta\tau) + 1}{2}\frac{sign(\beta T_s + \frac{1}{2} - \frac{1}{2}cos(id\theta) - \beta\tau) + 1}{2}, \tag{5d}$$

where $D'_A = \frac{D_A}{d^2 l_{BC}}$, $D'_B = \frac{D_B}{d^2 l_{BC}}$ and $D'_C = \frac{D_C}{d^2 l_{BC}}$, $\alpha = \frac{vk}{l_{BC}}$, $\beta = \frac{v}{dl_{BC}}$ and $d\theta = \frac{2\pi}{N}$ and $i$ ranged from 1 to $N$, the total number of points on the membrane. The boundary conditions of the equations are periodic. A differential equation describing the chemoattractant concentration, $I$, has been added to model its dynamics as the cell entered and exited the gradient. The *sign* functions are needed to account for the different levels of chemoattractant experienced by different points on the cell. For example, the front side of the cell, $\theta = 0$ experiences a higher chemoattractant level than the rear side, $\theta = \pi$. With the differential equations, we systematically simulate the dynamics for the selected set of parameters for $T_S = 10$ and $N = 20$ for a duration of $T_S + 20$.

We define the output signal for temporal sensing at each time point as the fold difference of the output (protein $C$) at that time from that at time $\tau = 0$ subtract by 1 (eqn.(6a)). The fold difference between the front half and back half of the cell reduced by 1 will be used to quantify the output response for spatial sensing (eqn.(6b)). The total output signal is obtained by integrating the output signal at each time point over time. Mathematically, the total output signal for the temporal sensing, $O_T$, and spatial sensing, $O_S$, are described as:

$$O_T = \frac{\int_0^\tau \sum_{i=1}^{N}(\frac{C_i(\tau)}{C_i(0)} - 1)d\tau}{N}, \tag{6a}$$

$$O_S = \int_0^\tau \frac{\sum_{i\in front} C_i(\tau)}{\sum_{i\in back} C_i(\tau)} - 1d\tau. \tag{6b}$$

To determine whether temporal or spatial sensing is better, we calculate the following ratio,

$$O_{TS} = \frac{O_T}{O_S}. \tag{7}$$

When $O_{TS} > 1$, the signaling output from temporal sensing is greater than that of spatial sensing and temporal sensing is favored as the sensing mechanism. Otherwise, spatial sensing is favored.

## Modelling noise in the external chemoattractant

To introduce noise into the external chemoattractant concentration, we solve a modified form of eqn.(5) using the Euler-Maruyama Method. The modified equations are as follows:

$$dA_i = (k'_{IA}I - l'_{FA}A + D'_A(A_{i+1} + A_{i-1} - 2A_i))d\tau, \tag{8a}$$

$$dB_i = \begin{cases} (k'_{IB}I - l'_{FB}B + D'_B(B_{i+1} + B_{i-1} - 2B_i))d\tau, & \text{(IFF), or} \\ (k'_{CB}C - l'_{FB} + D'_B(B_{i+1} + B_{i-1} - 2B_i))d\tau, & \text{(NFB)} \end{cases} \tag{8b}$$

$$dC_i = (k'_{AC}A\frac{1-C}{K_{AC}+1-C} - B\frac{C}{L_{BC}+C} + D'_C(C_{i+1} + C_{i-1} - 2C_i))d\tau \tag{8c}$$

$$dI_i = (\alpha\frac{sign(\frac{1}{2}cos(id\theta) - \frac{1}{2} + \beta\tau) + 1}{2}\frac{sign(\beta T_s + \frac{1}{2} - \frac{1}{2}cos(id\theta) - \beta\tau) + 1}{2})d\tau + \eta\alpha\sqrt{d\tau}Z(0,1), \tag{8d}$$

where $\eta$ quantified the fractional noise and $Z(0, 1)$ is the guassian distribution with mean 0 and variance 1.

To study the effects of noise on the sensing choice, we simulate the protein dynamics for the selected set of parameters for both the incoherent feedforward and integral feedback circuits as the cell moves through the noisy chemoattractant gradient for 10 times. We determine the values of $O_T$ and $O_S$ for each of the stochastic runs. If $O_T > 0$ and $O_S > 0$ for all the runs, both temporal and spatial sensing strategies could be used. In this case, temporal sensing is adopted if $\frac{O_T}{O_S} > 1$ and spatial sensing is adopted otherwise. For parameters where some runs yield $O_T < 0$ and all the runs yield $O_S > 0$, temporal sensing is infeasible and spatial sensing is adopted. Similarly, for parameters where some runs yield $O_S < 0$ and all the runs yield $O_T > 0$, spatial sensing is infeasible and temporal sensing is adopted. When some runs yield $O_T < 0$ and $O_S < 0$, sensing become infeasible using both

strategies. We determine the fraction of parameters that choose temporal versus spatial sensing as a function of $\eta$ for the following cases: (1) $\beta = 0.25$ , (2) $\beta = 1.0$ and (3) $\beta = 4.0$ at $D^{'}_{A} = 100$ and $D^{'}_{B} = 1$.

## Modelling noise in the internal signaling pathway

To introduce noise into the external chemoattractant concentration, we solve the following equations:

$$dA_i = (k^{'}_{IA}\xi I - l^{'}_{FA}\xi A + D^{'}_{A}(A_{i+1} + A_{i-1} - 2A_i))d\tau, \tag{9a}$$

$$dB_i = \begin{cases} (k^{'}_{IB}\xi I - l^{'}_{FB}\xi B + D^{'}_{B}(B_{i+1} + B_{i-1} - 2B_i))d\tau, & \text{(IFF), or} \\ (k^{'}_{CB}\xi C - l^{'}_{FB}\xi + D^{'}_{B}(B_{i+1} + B_{i-1} - 2B_i))d\tau, & \text{(NFB)} \end{cases} \tag{9b}$$

$$dC_i = (k^{'}_{AC}\xi A\frac{1-C}{K_{AC}+1-C} - \xi B\frac{C}{L_{BC}+C} + D^{'}_{C}(C_{i+1} + C_{i-1} - 2C_i))d\tau \tag{9c}$$

$$dI_i = (\alpha\frac{sign(\frac{1}{2}cos(id\theta) - \frac{1}{2} + \beta\tau) + 1}{2}\frac{sign(\beta T_s + \frac{1}{2} - \frac{1}{2}cos(id\theta) - \beta\tau) + 1}{2})d\tau, \tag{9d}$$

where $\xi = max(0, 1 + \nu Z(0, 1))$. In these equations, the rate constants are no longer fixed. They follow a normal distribution with their deterministic means and standard deviation given by, $\nu$. To prevent the rate constants from taking upon negative values, $\xi$ is given as the maximum of the rate constants and zero.

## Modelling other external chemoattractant profile

We also analyze the behavior of the system for a step change in external chemoattractant and an exponential varying external chemoattractant. In general, modeling a step change may be numerically unstable. We allow the step change to occur over 20 time steps and check that the system remains stable.

To model an exponential change in gradient, we modified eqn. 5(d) to become:

$$\begin{aligned} \frac{dI_i}{d\tau} =& \alpha exp(\frac{(\tau+1)(cos(id\theta)-1)}{2\beta})\frac{sign(\frac{1}{2}cos(id\theta) - \frac{1}{2} + \beta\tau) + 1}{2} \\ & \frac{sign(\beta T_s + \frac{1}{2} - \frac{1}{2}cos(id\theta) - \beta\tau) + 1}{2}, \end{aligned} \tag{10}$$
